# Prophylactic antineoplastic activity of Toxoplasma gondii RH derived antigen against ehrlich solid carcinoma with evidence of shared antigens by comparative immunoblotting

**DOI:** 10.1186/s13027-023-00500-3

**Published:** 2023-04-07

**Authors:** Maha M. Eissa, Maha R. Gaafar, Layla K. Younis, Cherine A. Ismail, Nahla El Skhawy

**Affiliations:** 1 Department of Medical Parasitology, Faculty of Medicine, Alexandria University, Alexandria, Egypt; 2 Department of Pathology, Faculty of Medicine, Alexandria University, Alexandria, Egypt; 3 Department of Clinical Pharmacology, Faculty of Medicine, Alexandria University, Alexandria, Egypt

**Keywords:** Immunomodulation, CD8^+^ T cells/Treg cells, VEGF, Angiogenesis

## Abstract

**Background:**

With cancer cases escalation, an urgent request to develop novel combating strategies arise. Pathogen-based cancer-immunotherapy is getting more consideration. Autoclaved parasitic antigens seem promising candidates, taking steadily their first steps. Our aim was to examine the prophylactic antineoplastic activity of autoclaved *Toxoplasma* vaccine (ATV) and to test for the shared antigen theory between *Toxoplasma gondii* and cancer cells.

**Methods:**

Mice were immunized with ATV followed by Ehrlich solid carcinoma (ESC) inoculation. Tumor weight, volume, histopathology, and immunohistochemistry for CD8^+^ T cells, Treg cells and VEGF were assessed. In addition, the proposed shared antigen theory between parasites and cancer was also verified using SDS-PAGE and immunoblotting.

**Results:**

Results revealed powerful prophylactic activity of ATV with 13.3% inhibition of ESC incidence, significant reduction in tumor weight and volume in ATV vaccinated mice. Immunologically, significantly higher CD8^+^T cells and lower FOXP3^+^ Treg cells surrounded and infiltrated ESC in ATV immunized mice with higher CD8^+^T/Treg cells ratio and significant antiangiogenic effect. Moreover, SDS-PAGE and immunoblotting showed four shared bands between Ehrlich carcinoma and ATV of approximate molecular weights 60, 26, 22 and 12.5 KDa.

**Conclusion:**

Exclusively, we demonstrated a prophylactic antineoplastic activity of autoclaved *Toxoplasma* vaccine against ESC. Moreover, to the best of our knowledge this is the first report highlighting the existence of cross-reactive antigens between *Toxoplasma gondi* parasite and cancer cells of Ehrlich carcinoma.

## Introduction

Since immune-suppression is one of cancer hallmarks, cancer-immunotherapy has emerged as a revolutionized weapon to fight cancer [1–3]. Unfortunately, upon using cancer antigens in immunotherapy, an immune tolerance is induced due to the familiarity of cancer antigens to the immune system. On the contrary, pathogens are foreign and could elicit stronger immune response, thus, could be promising candidates to cancer-immunotherapy trials [4–6].

In contrast to the acknowledged affiliation of some parasites as cancer promoters [4], low density chronic parasitic infection elicits a nonspecific antineoplastic activity through an immunomodulatory effect [5]. This harmonizes with the re-evaluated hygiene hypothesis that considered cancer as an outcome to lack of exposure to microorganisms during the childhood period (cancer hygiene hypothesis) [7].

In addition to the parasites’ immunomodulatory effect, shared antigens between parasites and cancer cells was postulated as another mechanism for the antineoplastic activity of parasites [5]. Antigens mainly glycoproteins, which are widely expressed in parasites can be detected in more than 80% of cancer patients as Thomsen Friedenreich (TF), GalNAc-Ser/Thr (Tn) antigens and others. These epitopes are immature glycosylation product of threonine and serine, naturally camouflaged by complete glycosylate chain and are strongly expressed on cancer cells [7]. They are also associated with disease metastasis and progression [8]. In human cancer cells specifically, mucin-type O-glycan structures are considered among the most specific cancer associated structures [9]. These mucin-type O-glycans proved to be shared with certain parasites as *Fasciola hepatica*, *Echinococcus granulosus*, and *Schistosoma mansoni*. This explains why the potent anti-cancerous properties are exhibited only by parasites rich in glycosylated antigens [5].

*Toxoplasma gondii* (*T. gondii*) is an intracellular apicomplexan pathogen that induces potent T helper 1 (TH1) response and antiangiogenic property [4]. The post-translational glycosylation of *Toxoplasma* tachyzoites are prevalent and large number of proteins with N and O-linked glycans are found in their secretory pathway favouring their potential shared antigens with cancer cells [10]. Furthermore, the enzyme essential for O-glycosylation has been characterized in *T. gondii* [11]. *Toxoplasma* demonstrated antineoplastic activity in some experimental studies against melanoma [12, 13], Lewis lung cancer [14], fibrosarcoma [15], sarcoma [16], pancreatic ductal adenocarcinoma [17], and Ehrlich ascites carcinoma (EAC) [18].

While using live pathogens to elicit an antineoplastic activity seems unrealistic, parasitic antigen are side way to hunt this activity. Autoclaved parasitic vaccines are special type of killed vaccines enclosing all the essential parasitic components. In addition, they are easy to prepare, safe, stable for long term storage, and potent immunostimulant [19]. In experimental trials, autoclaved vaccines provoked excellent protective capability against various parasitic infections including *Schistosoma mansoni* [20], *Trichinella spiralis* [21], *Leishmania donovani* [22], and *T. gondii* [23]. Interestingly, autoclaved parasitic vaccines provoked both an antineoplastic and immunomodulatory activity in experimentally induced cancer colon and arthritis in animal models, respectively [6, 24].

Consequently, in the current study, it seems noticeably worthy to investigate the prophylactic antineoplastic activity of ATV on a well-established cancer model as Ehrlich solid carcinoma (ESC). The proposed theory of shared antigens between parasites and cancer was also investigated between ATV and Ehrlich carcinoma.

## Material and methods

### Toxoplasma gondii strain and ATV preparation

Live *Toxoplasma gondii* tachyzoites of virulent RH HXGPRT (-) strain were maintained through serial intraperitoneal passages in Swiss albino mice [23]. ATV was prepared by autoclaving of collected tachyzoites at 120 °C, under pressure of 15 lb for 15 min [23, 25]. Autoclaved *Toxoplasma* tachyzoites were kept at − 20 °C until being lyophilized. Protein concentration was quantified using NanoDrop™ 2000 spectrophotometer (Thermo Scientific).

### Ehrlich ascites carcinoma cells

Ehrlich ascites carcinoma cells (EAC) in mouse were obtained from the National Cancer Institute (Cairo, Egypt). Cells were maintained through serial intraperitoneal passages of 2.5 × 10^6^ EAC cells diluted in sterile saline in a volume 0.2 ml in female Swiss albino mice [26].

### Animals

Thirty-six female Swiss albino mice 5–6 weeks old (20–25 g) and a two-kg male New-Zealand albino rabbit were allocated to this experiment. They were obtained from the animal house at the Department of Medical Parasitology, Faculty of Medicine, Alexandria University. Mice were housed under standard laboratory conditions (27 ± 2 °C; 70–80% humidity; 12 h light/dark cycle) with standard pellet diet and water ad libitum. Animals were handled in accordance to the ARRIVE guidelines for animal care and in compliance to the Institutional Animal Care and Use Committee in Faculty of Medicine, Alexandria University (IACUC, 0,201,396).

### In-vivo study of the prophylactic antineoplastic activity of ATV

Mice were randomly divided into two main groups. Group I: (21 mice) control group that was further subdivided into; subgroup Ia: (6 mice), served as normal control, subgroup Ib: (15 mice), served as Ehrlich solid carcinoma control (ESC control). Group II: (15 mice), served as ATV immunized, Ehrlich carcinoma cells inoculated group (ATV-ESC), where mice were immunized with two intradermal doses of 25 μg ATV over the sternum two weeks apart [27]. Then, two weeks after the second ATV dose, EAC cells were inoculated.

#### Induction of Ehrlich solid carcinoma in mice

To induce ESC in mice, EAC collected cells were counted and diluted in sterile saline. A volume of 0.2 ml containing 2.5 × 10^6^ EAC cells was injected subcutaneously on the back of each mouse [26]. Efforts were made to reduce animal suffering through daily observation and recording of pre-set humane endpoints including weight reduction, abnormal behaviors, labored breathing, lethargy, diarrhea, or abnormal mobility. Once an endpoint was reached, the animal was immediately euthanized and excluded from the study. On day 30 post-ESC induction [28], mice were anesthetized with thiopental sodium (45 mg/kg, intraperitoneal) and blood was collected for biochemical analysis. After euthanization by an overdose of thiopental, solid tumors and livers were carefully excised.

#### Hepatic transaminases

To estimate the hepatic impact of both ESC and ATV immunized, Ehrlich carcinoma cells inoculated mice, liver transaminase enzymes were assessed. Aspartate aminotransferase (AST) and Alanine aminotransferase (ALT) were measured using chemical auto-analyser Dimension RxL Max (Siemens Health Care Diagnostics, USA).

#### Pathological examination

Ehrlich solid tumors were weighted, measured, and tumor volumes were calculated using the following equation: ***1/2 × L x W x H,*** Where, L is length, W is width and H is height and expressed in mm^3^ [16]. Excised samples of livers and ESC were fixed in 10% buffered formalin, processed, and embedded in paraffin and sections were stained by haematoxylin and eosin (H&E) staining for histopathological examination. A semiquantitative scoring system was used to assess hepatic histopathological changes, as described with some modification [29]. Sections of ESC were additionally stained by Masson Trichrome staining to assess degree of fibrosis. Both fibrosis and necrosis were semiquantitatively assessed and graded as previously described with modification [30, 31].

##### Immunohistochemistry

Sections of ESC were subsequently stained by immunohistochemistry (IHC) for analysis of cytotoxic T cells (CD8^+^) T cells, forkhead box P3^+^ (FOXP3^+^) regulatory T cells (Treg), and vascular endothelial growth factor (VEGF) using horseradish peroxidase (HRP). The following monoclonal antibodies were used per manufacturer guidance: Anti-CD8 (Ab-1) monoclonal antibody (Thermoscientific), to assess the intensity of CD8^+^ T cell subset surrounding and infiltrating tumor tissue. Anti-FOXP3 (86 D) monoclonal antibody (BioCare Medical) to assess the intensity of FOXP3 Treg cells surrounding and infiltrating tumor tissue. Anti-VEGF polyclonal antibody (BioGenex) to assess angiogenesis. Sections were deparaffinized and washed with buffer. Antigen retrieval was performed using 10 mM sodium citrate buffer of PH 6.0 for 10 min at 80 °C. Three % hydrogen peroxide was used for 10 to 15 min to block endogenous peroxidases followed by washing 4 times with buffer. Slides were stained using the three tested primary antibodies separately according to the manufacturer protocol. Then, slides were washed and incubated with HRP-conjugated secondary antibody (UltraVison ONE HRP Polymer, Thermoscientific) for 30 min at room temperature in a dark place followed by washing with buffer. Slides were visualized by peroxidase compatible chromogen, washed, and finally counterstained. For each IHC run, a positive control for each antibody was performed. For VEGF, angiosarcoma sections were used while for FOXP3 and CD8, sections of tonsils or lymph nodes were used as positive control. Negative controls were included by omission of the primary antibody. A semi-quantitative analysis was performed according to the number of positively stained cells / HPF [6, 28].

### Proteomic study and immunoblotting

#### Rabbit anti-Toxoplasma hyperimmune serum

To test for shared antigens between Ehrlich carcinoma, and ATV, rabbit anti-*Toxoplasma* hyperimmune serum was prepared. The rabbit was injected subcutaneously with 0.5 mg of ATV in sterile isotonic saline. Three booster doses were given at two weeks interval. Two weeks after the last injection, the rabbit was exsanguinated and serum was separated, then stored at − 20 °C for later use [32]. Rabbit anti-*Toxoplasma* serum IgG was measured using ARCHITECT i1000SR Immunoassay (Abbott, USA).

#### Proteomic study and immunoblotting

Lysate of Ehrlich carcinoma were prepared as previously described [33]. Protein concentration was quantified using NanoDrop™ 2000 spectrophotometer (Thermo Scientific) SDS-PAGE (Laemmli) was conducted for Ehrlich carcinoma lysate and ATV, as described previously [34, 35]. PageRuler Plus Prestained Protein Ladder (Thermo Scientific) and samples were allowed to run using an 8% stacking gel (Tris–HCl PH:6.8) followed by 12% resolving gel (Tris–HCl PH:8.8). Gel was stained with Commasie Brilliant blue R 250 (Sigma-Aldrich), documented using Gel Doc™ XR + (BIO-RAD) documentation system and analysed by Image Lab software 5.1 (BIO-RAD).

Immunoblotting was performed using rabbit IgG DAB Chromogenic Reagent Kit (Blue) (Boster Biological Technology Co., Ltd) [34]. Ehrlich carcinoma lysate, and ATV were separated on SDS-PAGE gel, then transferred to a nitrocellulose membrane overnight. The membrane was blocked, washed, and incubated with the diluted rabbit hyperimmune sera (1:100) for 1 h at 20–30 °C, then at 4 °C overnight with agitation. The membrane was then washed and incubated with diluted goat anti-rabbit secondary antibody (1:2000) at 20–30 °C for 90 min with agitation. Finally, it was washed, and chemiluminescent detection was performed. It was documented using Gel Doc™ XR + (BIO-RAD) documentation system.

### Statistical analysis

Data presented is the average of two experimental replicate. Statistical analysis was performed using IBM® SPSS® Statistics, version 25. Quantitative data was analysed with descriptive analysis and expressed as mean ± standard error of the mean (SEM). Independ-t test was used to compare between two quantitative variables. ANOVA with post-hoc Tukey’s test was used to compare more than two quantitative variables. Kruskal Wallis was used to analyse qualitative data and significance was adjusted by Bonferroni correction for multiple tests. Significance was considered when *p* values were < 0.05. Graphs were plotted using GraphPad Prism®, version 8.0.2 with representation of the mean and SEM.

## Results

### Hepatic transaminases

**Fig. 1:**
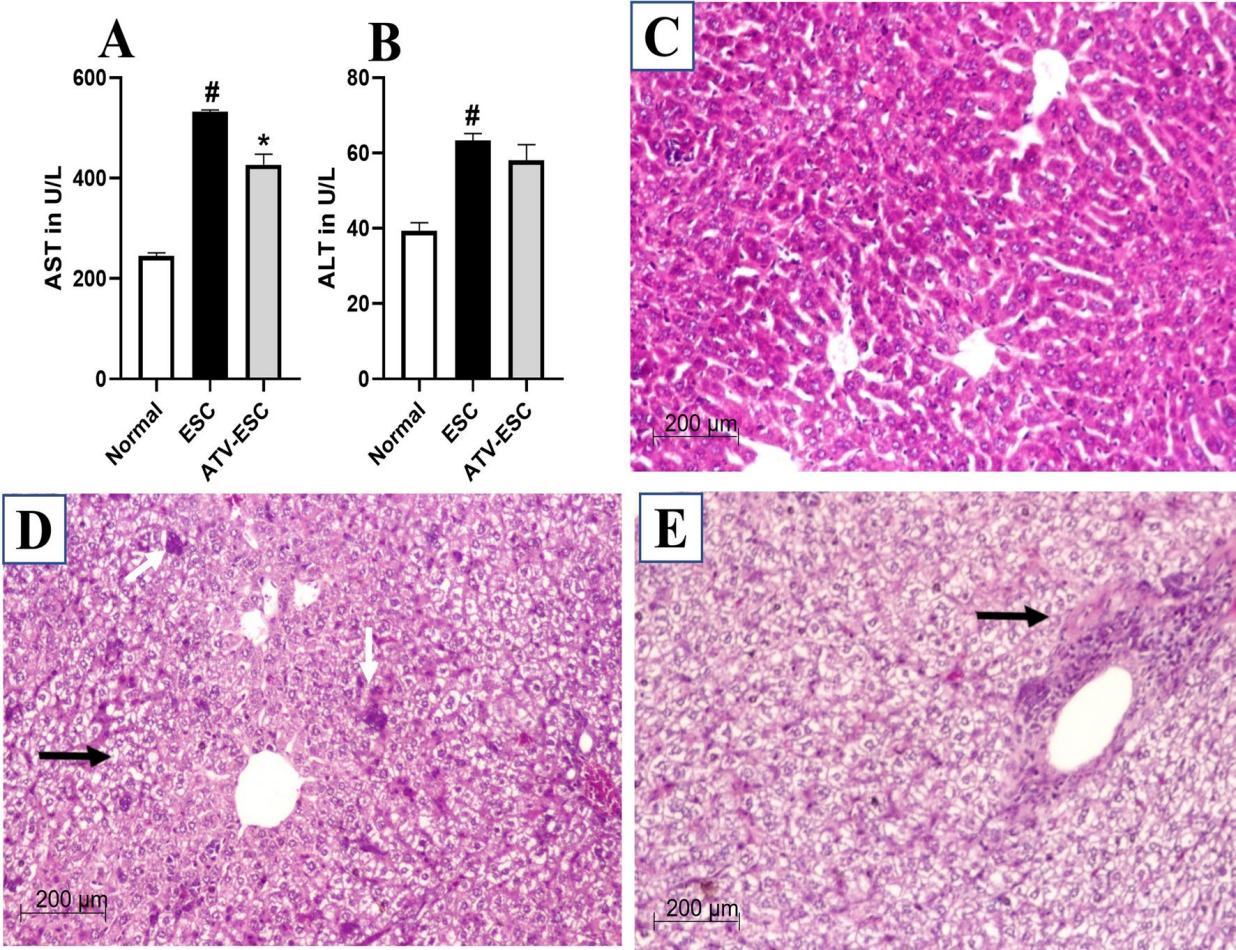
Hepatic transaminases and representative photomicrographs of H&E-stained liver sections. Mean ± SEM of liver enzymes (**A**: AST, **B**: ALT in U/L) measured in different groups, Normal: Normal control group, ESC: ESC control group, ATV-ESC: ATV immunized, Ehrlich carcinoma cells inoculated group. AST: Aspartate aminotransferase ALT: Alanine aminotransferase: #*p* < 0.05 compared to normal control (*p* < 0.05). *p < 0.05 compared to ESC control. **C**–**E** Liver sections of different studies groups **C**: Normal control showing preserved liver architecture (H&E) **D**: ESC control showing diffuse fatty changes (black arrow) with mild inflammatory aggregates (white arrow) (H&E), **E**: ATV immunized, Ehrlich carcinoma cells inoculated group showing fatty changes with moderate lymphocytic aggregates in the portal tract (black arrow) (H, E). Scale bar = 200 μm

As shown in Fig. 1A, B, liver enzymes (AST and ALT, respectively) of ESC control were significantly elevated compared to the normal control (Ia) (*p* < 0.05). On the other hand, liver enzymes were reduced in ATV immunized, Ehrlich carcinoma cells inoculated group compared to the ESC control, yet significance was only observed for AST (*p* < 0.05).

### Hepatic histopathological examination

In normal control, hepatic H&E staining sections showed a preserved architecture with normal looking hepatic cord and sinusoidal spaces, with no inflammatory cells in the portal tracts (Fig. 1C). Liver sections from ESC control revealed diffuse fatty changes and mild periportal inflammatory aggregates (Fig. 1D). Whereas liver sections from ATV immunized, Ehrlich carcinoma cells inoculated group revealed fatty changes with moderate lymphocytic aggregates in the portal tract (Fig. 1E).

### Ehrlich solid carcinoma

#### Gross pathological findings

**Fig. 2:**
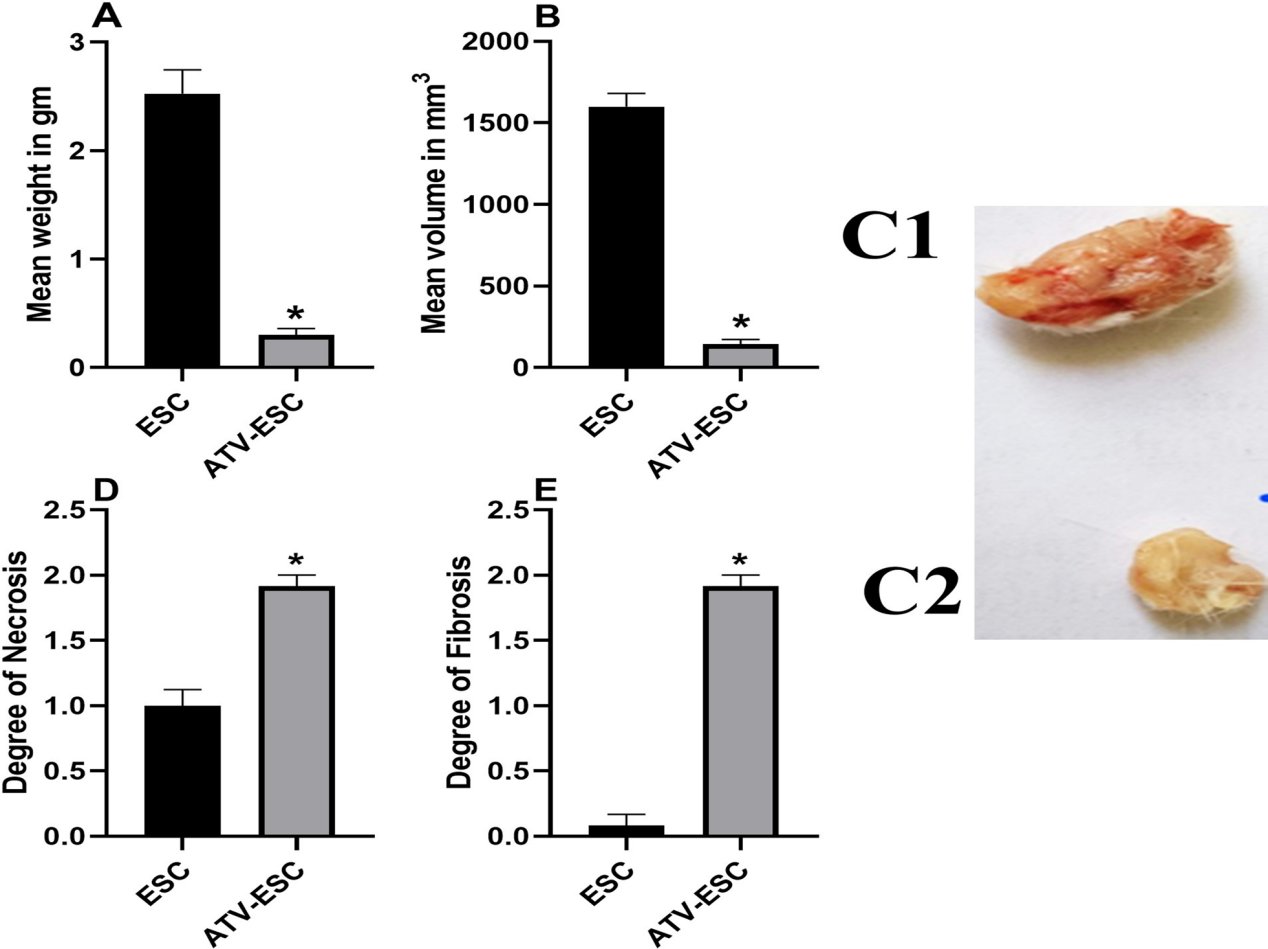
Characteristics of Ehrlich solid carcinoma and representative gross pathological pictures of Ehrlich solid carcinoma. Effect of ATV immunization on mean ± SEM of weight (**A**) and mean volume (**B**) of ESC in the different studied groups, ESC: ESC control, ATV-ESC: ATV immunized, Ehrlich carcinoma cells inoculated group. **C** Gross appearance of ESC from the different studied groups,** C1**: ESC from ESC control,** C2**: ESC from ATV immunized, Ehrlich carcinoma cells inoculated group. Effect of ATV immunization on mean ± SEM of necrosis (**D**) and fibrosis (**E**) of ESC in the different studied groups *p < 0.05 compared to ESC control

All mice in ESC control developed ESC, while only 13/15 mice in ATV immunized, Ehrlich carcinoma cells inoculated group developed ESC with a inhibition rate of 13.3%. Both ESC weight and volume from ATV immunized, Ehrlich carcinoma cells inoculated group were significantly reduced versus those of ESC control (Fig. 2A–C) (*p* < 0.05).

#### Histopathological findings

**Fig. 3:**
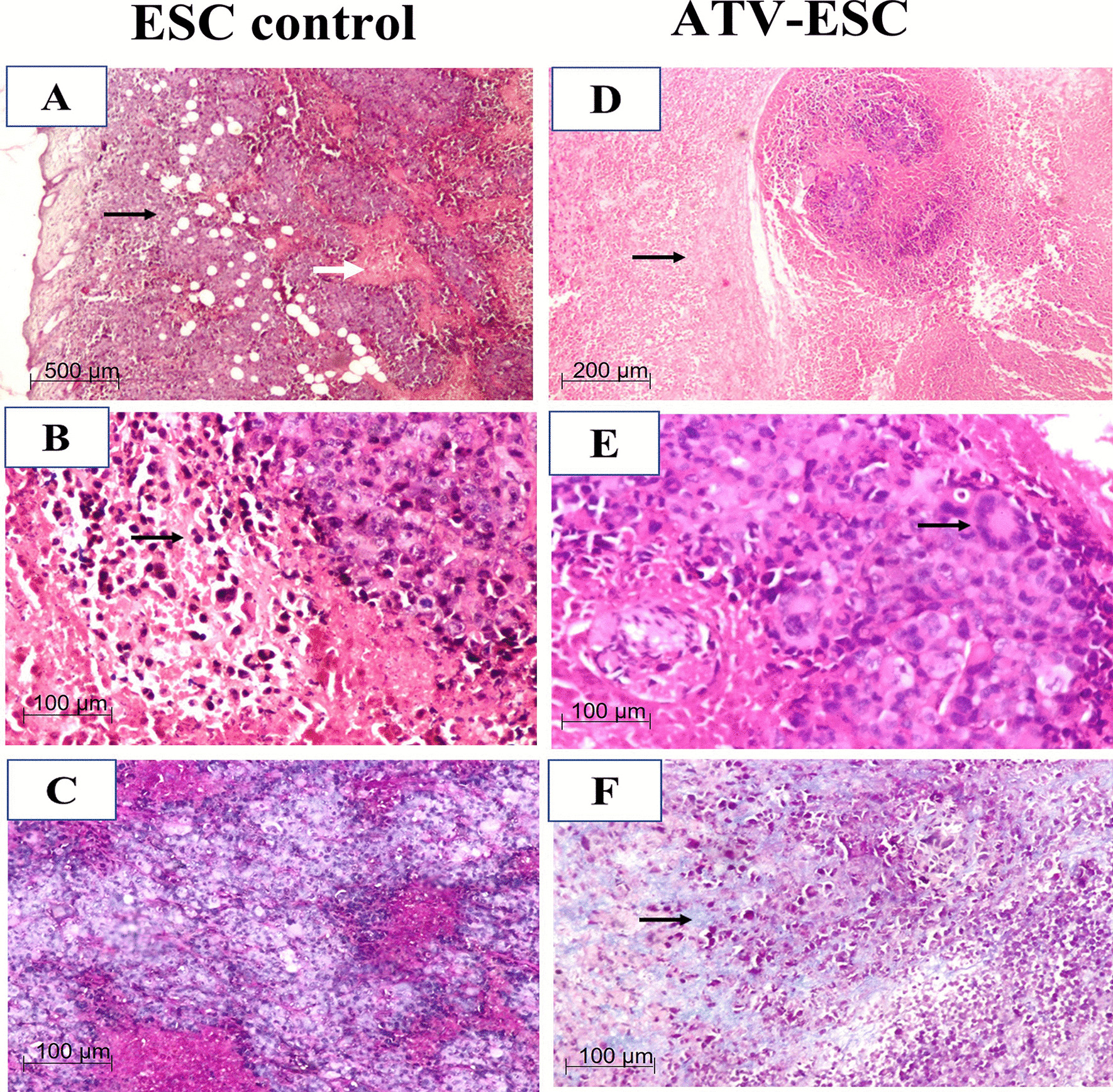
Representative photomicrographs of H & E and Masson Trichrome stained Ehrlich solid carcinoma sections of the different studied groups. Histopathological finding in ESC from ESC: ESC control, ATV-ESC: ATV immunized, Ehrlich carcinoma cells inoculated group. Sections of ESC from ESC control group showing: **A** shapes of tumor cells invading subcutaneous fat (black arrow) with areas of mild necrosis (white arrow) (**H**&**E**), Scale bar = 500 μm, **B** High mitotic activity (black arrow) (**H**&**E**), Scale bar = 200 μm, **C** no fibrosis (Masson Trichrome staining). Sections of ESC from ATV immunized, Ehrlich carcinoma cells inoculated group showing **D**: more necrosis (black arrow) with vanishing tumor cells (**H**&**E**), **E** granulomas with giant cells (black arrow) (**H**&**E**), **F** moderate degree of fibrosis (black arrow) (Masson Trichrome staining). Scale bar = 100 μm

In ESC control, tumor sections revealed subcutaneous sheets of highly malignant cells with pleomorphic hyperchromatic nuclei, increased nucleocytoplasmic ratio, and numerous mitotic figures (Fig. 3A, B). Areas of mild necrosis (grade 1) were also noted (Fig. 2D). On the other hand, ESC from ATV immunized, Ehrlich carcinoma cells inoculated group showed more necrosis (grade 2) that was significantly higher compared to ESC control (Fig. 2D) (*p* < 0.05) Remarkable reduction in tumor cells with giant cells infiltration were detected in some examined samples (Fig. 3D, E). Sections stained with Masson Trichrome stain showed no fibrosis (grade 0) in the ESC control, whereas in ATV immunized, Ehrlich carcinoma cells inoculated group, there was moderate fibrosis (grade 2) that was significantly higher compared to ESC control (Figs. 2E, 3C&F, respectively) (*p* < 0.05).

#### Immunohistochemical analysis

**Fig. 4:**
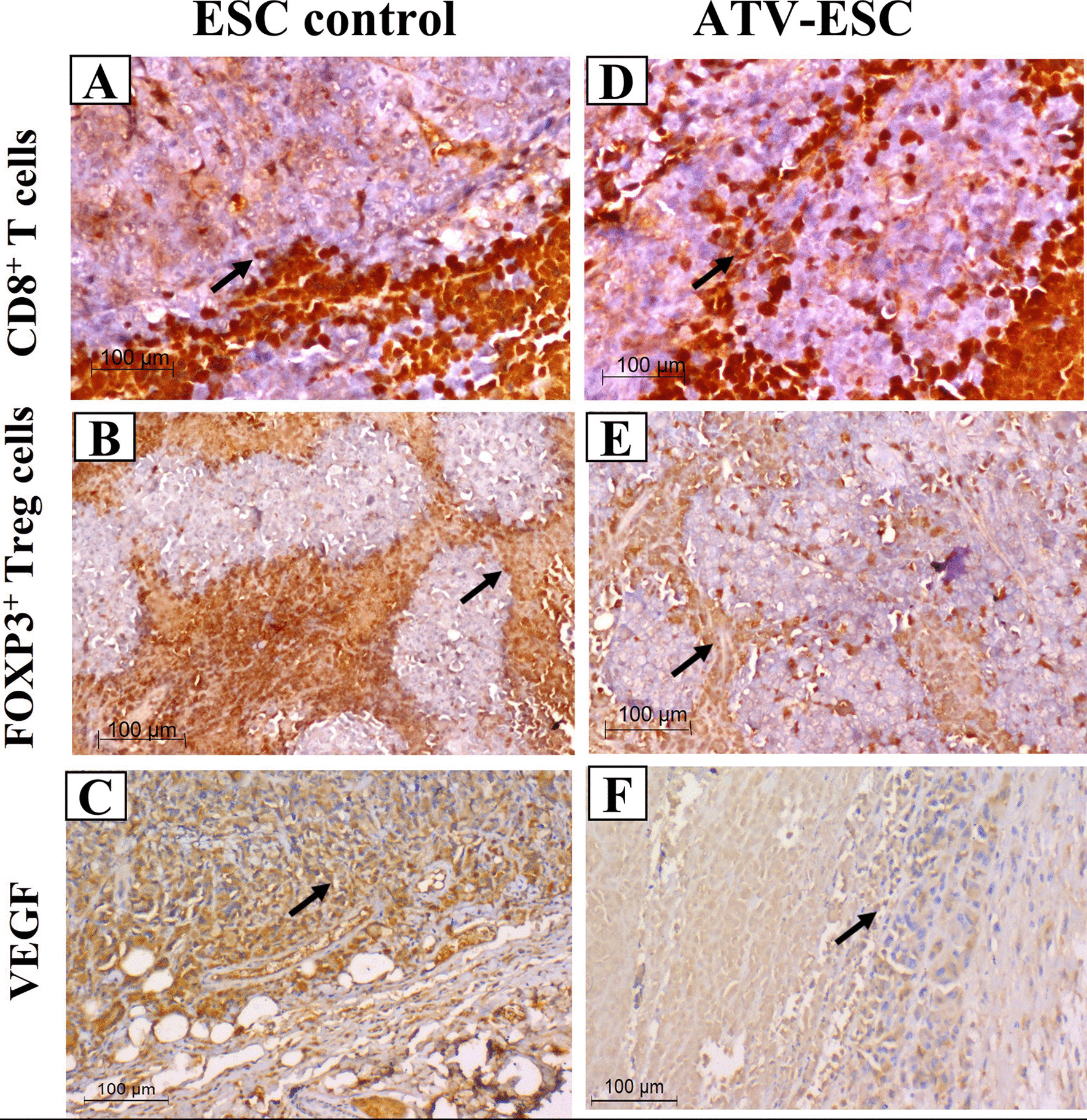
Representative photomicrographs of IHC stained immune cells and VEGF expression in Ehrlich solid carcinoma for CD8^+^ T cells, FOXP3^+^ Treg cells and VEGF. IHC finding in ESC from ESC: ESC control, ATV-ESC: ATV immunized, Ehrlich carcinoma cells inoculated group. Stained sections of ESC from ATV immunized, Ehrlich carcinoma cells inoculated group showed abundant CD8^+^ T cells, fewer FOXP3^+^ Treg surrounding and infiltrating tumor tissue (**A**, **B**, **D**, **E**) and less VEGF (**C**, **F**) compared to ESC sections from control group. Black arrow showing positively stained immune cells (IHC). Scale bar = 100 μm

**Fig. 5:**
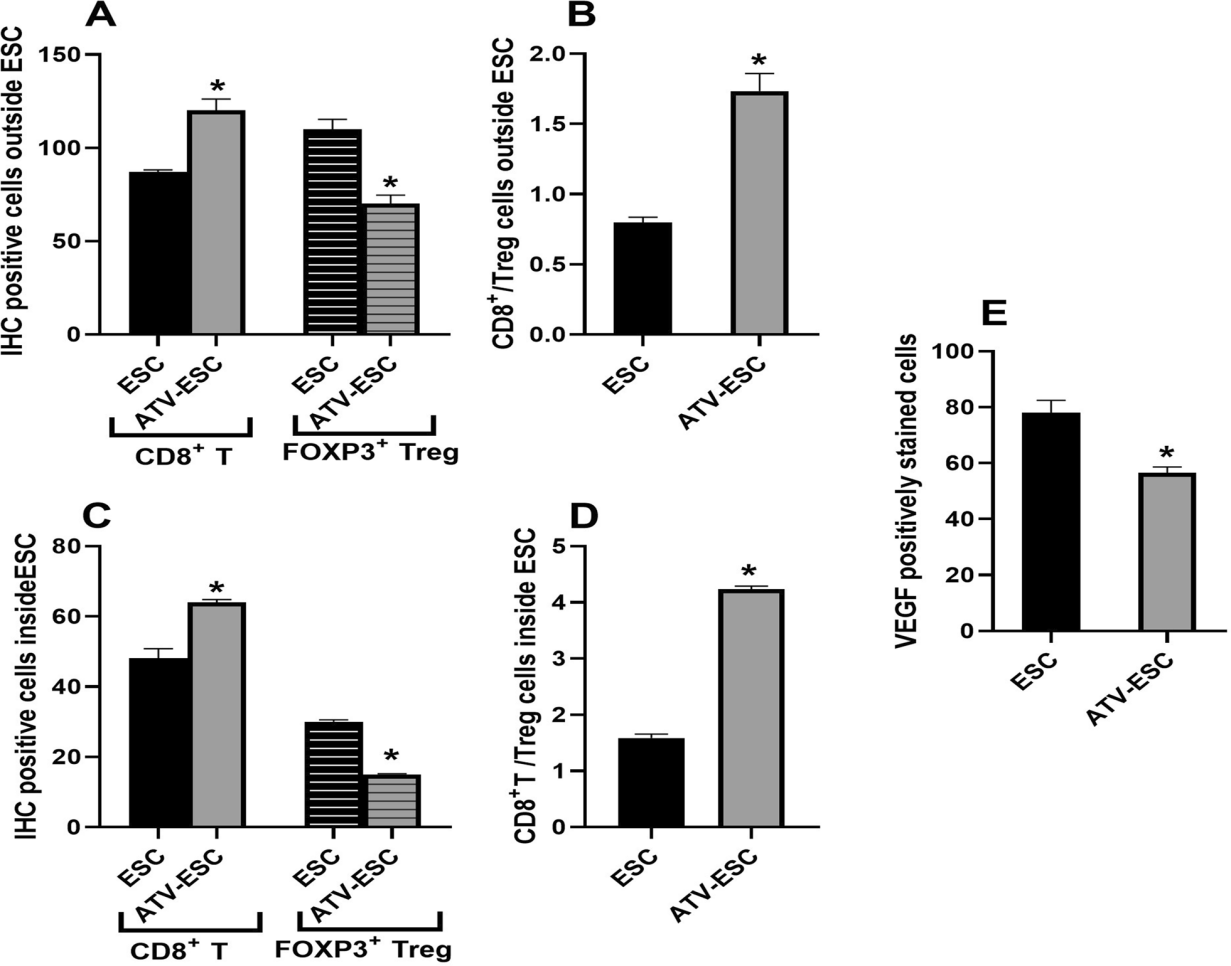
Graphs of the IHC positively stained immune cells (CD8^+^ and FOXP3^+^ Treg cells) and VEGF expression. Mean ± SEM of immune stained cells outside (**A**) and inside ESC (**C**) and their ratio (**B**, **D** respectively) in ESC sections from ESC: from ESC control group and ATV-ESC: ATV immunized, Ehrlich carcinoma cells inoculated group. (**E**) Mean VEGF in ESC from ESC control group and ATV immunized, Ehrlich carcinoma cells inoculated group. *:* p* < 0.05 compared to ESC control

As shown in Fig. 4A, B, D, E, analysis of immune cells surrounding, and infiltrating ESC tissues revealed significantly higher CD8^+^ T cells and lower FOXP3 ^+^ Treg cells in ATV immunized, Ehrlich carcinoma cells inoculated group compared to ESC control (Fig. 5A, C) (*p* < 0.05). The ratio of CD8^+^ T cells/Treg cells surrounding, and infiltrating ESC was significantly higher in ATV immunized, Ehrlich carcinoma cells inoculated group versus ESC control (Fig. 5B, D) (*p* < 0.05). To examine angiogenesis, ESC tumor sections were stained for VEGF, as shown in Fig. 4C, F. Statistically, a significant reduction of VEGF was detected in ESC sections from ATV immunized, Ehrlich carcinoma cells inoculated group versus ESC control (Fig. 5E) (*p* < 0.05).

### Proteomic study

**Fig. 6:**
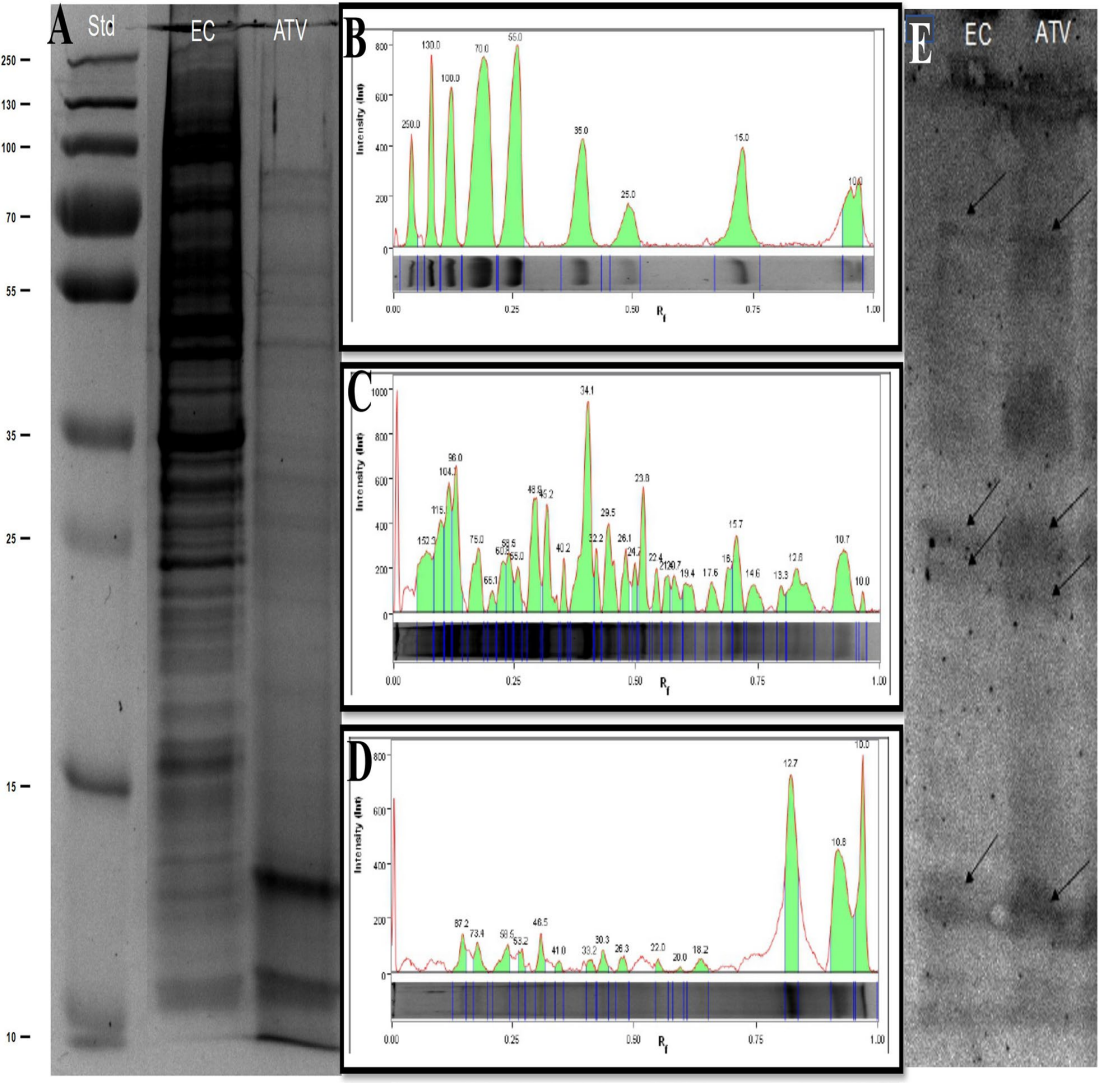
Proteomic analysis and immunoblotting (**A**) SDS-PAGE (Laemmli) gel, 8% stacking gel, 12% resolving gel, stained with Commasie Brilliant blue showing: standard (Std), Ehrlich carcinoma (EC) and Autoclaved *Toxoplasma* vaccine (ATV). (**B**–**D**) Lane profile anlaysis of standard, EC and ATV respectively. (**E**) Immunoblotting of Ehrlich carcinoma (EC) and Autoclaved *Toxoplasma* vaccine (ATV) incubated with rabbit anti-*Toxoplasma* hyperimmune sera. Shared bands are pointed at by black arrow

SDS-PAGE analysis of Ehrlich lysate showed the existence of 30 protein bands with molecular weights range from 152–10 KDa (Fig. 6A, B). The highest band intensity was observed at band (3^rd^, 4^th^, 13^th^) of approximate molecular weight 104.2, 96 and 34.1 KDa (Fig. 6C). On the other hand, only 15 protein bands were detected in ATV with molecular weights range from 87–10 KDa. Bands (13th 14th and 15^th^) of approximate molecular weight 12.5, 10.8 and 10 KDa respectively showed the highest intensity (Fig. 6D). Multiple bands of similar molecular weights were noticed between Ehrlich carcinoma, and ATV. Using immunoblotting to verify the theory of shared antigens revealed attachment of *Toxoplasma* antibodies to four protein bands of approximate molecular weights 60, 26, 22, and 12.5 KDa in both Ehrlich carcinoma and ATV lanes (black arrows in Fig. 6E).

## Discussion

In the battle towards discovery of a cancer vaccine, scientists are chasing safe, effective, and natural-based candidates. Their aim is to prompt a strong long-term immunity and establish an immune memory, hence, putatively prevent occurrence of cancer and limit the recurrence of an already excised tumor mass [8]. Pathogen-based cancer therapy appears in the horizon as the savior.

Helminth and protozoa enlightened the path for their antineoplastic activity. Autoclaved *Schistosoma mansoni* antigen showed therapeutic antineoplastic efficacy against colon cancer in mice [6]. *T. cruzi* epimastigote lysate antigen promoted antineoplastic activity against colon and breast cancer in rats [36]. Interestingly, *T. gondii* fulfills criteria in cancer vaccine checklist as low titer of antibodies was related to cancer resistance [37]. Furthermore, *Toxoplasma* antibodies successfully attached to mouse melanoma and breast cancer cell lines [32]. In addition, the abundance of glycosylated antigens in *T. gondii* reinforces the argument to enroll it to fight cancer [10].

Autoclaved parasitic vaccines have long been considered as a safe, stable, easy to prepare and cheap choice for active immunization with potent immunogenic potency against their corresponding parasitic infection [19, 20]. ATV promoted excellent protective efficacy against experimental toxoplasmosis with TH1 immune stimulation, abundance of splenic CD8^+^ T cells and 78.8% of reduction of parasite load in spleen compared to their control, superior to *Toxoplasma* lysate antigen [23]. Therefore, investigating the prophylactic potential of ATV as well as the proposed theory of shared antigens between parasites and cancer can be an initiative step to enroll parasite-based cancer vaccines to cancer immunotherapy list.

During *in-vivo* studies in different cancer models, remarkable elevation in liver enzymes was usually noticed, induced by cancer cells that disrupt hepatic metabolism and destroy hepatocytes [28, 38]. In our study, both AST and ALT were significantly elevated in ESC control compared to normal control, highlighting a sort of liver insult, in line with previous studies [26]. Immunization with two doses of ATV prior to induction of ESC reduced liver enzymes indicating a prospective ATV hepatic ameliorative role in ESC mice.

Amid the elevated liver enzymes in ESC control, hepatic histopathological examination revealed diffuse fatty changes associated with periportal inflammatory aggregates. This matches with previous studies reporting hepatic fatty changes as pathological insult induced in ESC model [28]. Upon immunization with ATV, aggregates of lymphocytes paved their way around the periportal tract, denoting a sort of immune-mediated hepatitis. This was described previously in cancer patients with solid tumors receiving immunotherapy. Those patients are at higher risk of developing different grades of hepatitis, surprisingly without blood signs of hepatotoxicity, which fits with our findings [39].

In the present *in-vivo* study, immunization with two doses of ATV prior to inoculation of EAC reduced the incidence of ESC development by 13.3% denoting a promising role of ATV as a prophylactic cancer vaccine candidate. In conjugation with these findings, vaccination with lysate of epimastigote stage of *T. cruzi* inhibited the development of chemically induced breast and colon cancer in rat models [36].

Besides, ATV immunized, Ehrlich carcinoma cells inoculated mice showed statistically significant marked reduction in ESC weight and volume versus the ESC control. Previous reports revealed similar responses using different *Toxoplasma* varieties. Prophylactic use of gamma irradiated attenuated cysts of *Toxoplasma* significantly inhibited Ehrlich ascites carcinoma cell proliferation, invasion and promoted cell apoptosis [18]. Correspondingly, intratumorally injected attenuated *Toxoplasma* induced potent reduction in melanoma size in mice [12]. *Toxoplasma* tachyzoites that were frequently frozen-thawed and lysate antigen decreased size and weight of fibrosarcoma and sarcoma in mice, respectively [15, 16]. Some helminths as well induced reduction in tumor sizes in different cancer models including live *Trichinella spiralis*, hydatid cyst fluid against melanoma models [40, 41] and autoclaved *Schistosoma mansoni* antigen against cancer colon in murine models [6]. The significant reduction of ESC weight and volume was coupled with enhancement of liver enzymes in ATV immunized, Ehrlich carcinoma cells inoculated mice, as reported before [26, 28].

Pathological examination of ESC sections from ESC control showed sheets of malignant tumor cells in line with previous studies [26]. On the other hand, more necrosis, infiltration of giant cells and moderate degree of fibrosis were noted in ESC sections in ATV immunized, Ehrlich carcinoma cells inoculated mice compared to ESC control, implying sort of immune battle as well as impairment of blood supply taking place, later investigated using IHC.

These results match with previous conclusions reporting central necrosis encountered in untreated tumors as a poor prognostic criterion probably due to the release of proinflammatory mediators that stimulate angiogenesis and tumor proliferation [42]. Upon immune stimulation with immunotherapeutic agents, necrosis is noted as a positive prognostic criterion, since the released contents of necrotic cell promote activation of cytotoxic T cells and antigenic presentation [43]. Fibrosis as well is a tumor prognostic criterion since it is considered a good prognostic factor favouring tumor encapsulation following cancer immunotherapeutic modalities denting sort of tissue healing [44].

Dominance of immune suppression is one of cancer hallmarks mediated via myeloid-derived suppressor cells that produce high levels of tumor growth factor (TGF). Within the tumor microenvironment, TGF selectively attracts Treg cells and promote an in-situ transformation of CD4^+^ T cells into suppressive T cells (Treg). Those attracted Treg cells have higher immunosuppressive capacity compared to normal Treg cells [45, 46]. In fact, present data revealed high level of Treg cells surrounding and infiltrating ESC in ESC control. Following ATV immunization, depletion of Treg cells both outside and inside the ESC tumor tissue was noted, denoting liberation from the immunosuppressive dominance. Remarkably, fascinating arrival of cytotoxic T cells in the tumor microenvironment took place.

Crawling of cytotoxic CD8^+^ T cells around and inside tumor tissues is a promising indicator of establishment of immune stimulation and linked to antineoplastic activity in multiple models using *Toxoplasma* and *T. cruzi* antigens [12, 17, 36, 47]. Hence, studies highlighted the value of the ratio between CD8^+^ effector T cells/Treg cells infiltrating the tumour being the most crucial prognostic factor [2, 45]. This was evidenced, herein, by successful pattern of immune stimulation both surrounding and infiltrating the tumor tissue, revealed by IHC. It was represented as a significant elevation of CD8^+^ T cells and depletion of Treg cell with higher CD8^+^/Treg cells ratio both surrounding and infiltrating ESC.

Aside from the immunomodulatory effect, and in severely compromised immunodeficiency (SCID) mice, *Toxoplasma* is still able to elicit an antineoplastic activity via an antiangiogenic capacity promoting significant hypoxia [13]. An essential tool for tumor growth and metastasis is neoangiogenesis mediated through VEGF. Deprivation from blood supply is a magic strategy that attracted oncologist to induce tumor cell death [1]. Our results reported significant reduction in VEGF, main proangiogenic factor, in ESC from ATV immunized, Ehrlich carcinoma cells inoculated mice. These data conjugate with previous reports highlighting the antiangiogenic property of *Toxoplasma* in different cancer models [14, 18]. Additionally, VEGF is a powerful promoter of immunosuppression that directly correlates with Treg population while inversely corelates with CD8^+^ cytotoxic T cell population [48]. These data fit within our results since immunization with ATV promoted stimulation of the immune system with suppression of both VEGF and Treg cell population while crawling of CD8^+^ cytotoxic cells within ESC.

While the immunomodulatory and antiangiogenic potency of ATV has been investigated, the additional theory of shared antigens between ATV and cancer cells was next to be considered. Shared antigens and their cross reactivity were confirmed between different parasites and cancer cells [5, 49–51]. Mucin-type O-glycan structures (Tn, sial Tn, TF, Tk) are among the most specific cancer associated antigens, which are vital in malignant cell invasion and metastasis. Interestingly, these antigens have been reported to be expressed in different parasites [5, 9, 52]. In *Toxoplasma gondii*, the enzyme essential for mucin type O-glycosylation has been characterized favoring its potential shared antigens with cancer cells [11].

Upon conducting SDS-PAGE on Ehrlich carcinoma lysate and ATV, multiple bands of similar molecular weights have been detected. Our data is in line with data confirming the abundance of low molecular weight immunogenic proteins derived *from Toxoplasma gondii* RH stain ranging from 93–12 KDa [53–55]. For further verifying shared antigens, immunoblotting was performed using anti *Toxoplasma* hyperimmune sera. Interestingly, *Toxoplasma* antibodies were able to bind to four proteins in both Ehrlich carcinoma lysate and ATV of approximate molecular weights 60, 26, 22, and 12.5 KDa suggesting shared antigens between Ehrlich carcinoma and ATV and further supporting the molecular mimicry theory raised between parasites and cancer.

## Conclusion

Pathogen-based cancer-immunotherapy is getting more consideration. Autoclaved *Toxoplasma* vaccine has shown potent prophylactic antineoplastic activity against ESC through an immunomodulatory and antiangiogenic effect with existence of shared antigens between Ehrlich carcinoma and ATV. These promising results may create a paradigm shift in cancer vaccination. Further studies are ongoing to examine the role of autoclaved parasitic vaccines against different cancer models. Furthermore, characterization and identification of the shared antigens between parasites and cancer is a noteworthy step to open the gate for the discovery and the development of highly immunogenic cancer vaccine candidates of parasitic origin, thus enrich the cancer immunotherapy list for the incurable cancer disease.

## Data Availability

The dataset(s) supporting the conclusions of this article is(are) included within the article.

## Abbreviations

ATV: Autoclaved *Toxoplasma* vaccine
ESC: Ehrlich solid carcinoma
*T. gondii*: *Toxoplasma gondii*
Treg: T regulatory cells
VEGF: Vascular endothelial growth factor
